# Timing HIV infection with a simple and accurate population viral dynamics model

**DOI:** 10.1098/rsif.2021.0314

**Published:** 2021-06-30

**Authors:** Daniel B. Reeves, Morgane Rolland, Bethany L. Dearlove, Yifan Li, Merlin L. Robb, Joshua T. Schiffer, Peter Gilbert, E. Fabian Cardozo-Ojeda, Bryan T. Mayer

**Affiliations:** ^1^ Vaccine and Infectious Diseases Division, Fred Hutchinson Cancer Research Center, Seattle, WA, USA; ^2^ US Military HIV Research Program, Walter Reed Army Institute of Research, Silver Spring, MD, USA; ^3^ Henry M. Jackson Foundation for the Advancement of Military Medicine, Bethesda, MD, USA; ^4^ Department of Medicine, University of Washington, Seattle, WA, USA; ^5^ Department of Statistics, University of Washington, Seattle, WA, USA

**Keywords:** HIV, viral dynamics, mathematical modelling, infection timing, clinical trials

## Abstract

Clinical trials for HIV prevention can require knowledge of infection times to subsequently determine protective drug levels. Yet, infection timing is difficult when study visits are sparse. Using population nonlinear mixed-effects (pNLME) statistical inference and viral loads from 46 RV217 study participants, we developed a relatively simple HIV primary infection model that achieved an excellent fit to all data. We also discovered that Aptima assay values from the study strongly correlated with viral loads, enabling imputation of very early viral loads for 28/46 participants. Estimated times between infecting exposures and first positives were generally longer than prior estimates (average of two weeks) and were robust to missing viral upslope data. On simulated data, we found that tighter sampling before diagnosis improved estimation more than tighter sampling after diagnosis. Sampling weekly before and monthly after diagnosis was a pragmatic design for good timing accuracy. Our pNLME timing approach is widely applicable to other infections with existing mathematical models. The present model could be used to simulate future HIV trials and may help estimate protective thresholds from the recently completed antibody-mediated prevention trials.

## 1. Introduction

A key challenge for HIV prevention trials is dating the exposure that ultimately led to breakthrough infection. The estimation of infection time subsequently allows the inference of the concentration of the protective agent at exposure, which is critical to understanding why HIV acquisition was not prevented. Early infection is difficult to study in practice; even if prospective sampling were available, HIV RNA is not detectable in blood during early HIV infection and not all participants can accurately point to potential recent exposure events. Therefore, to estimate the time of infection—or the eclipse phase, the period between HIV acquisition and first detectable viral load—a model or inference technique is required.

Estimation techniques have been described previously. Several use viral sequence data and evolutionary models to trace time back to the founder sequence [1–4]. Others use viral load data prior to viral peak and retrace using log-linear regression (average or maximum upslope) [3]. Mathematical models of viral load have also been used for timing HIV infection [5] (and SARS-CoV-2 [5]). Still other approaches apply diagnostic windows leveraging Fiebig staging [6] and prior knowledge of eclipse-phase duration [7,8]. Finally, combinations of some of these approaches have been integrated into a comprehensive statistical framework [9].

Population nonlinear mixed-effects (pNLME) modelling is a powerful tool to estimate mechanistic model parameters from longitudinal data across individuals [10,11]. It has been used extensively in pharmacokinetic modelling [12,13] and, in recent years, increasingly applied to viral dynamics [14–19].

The RV217 study [20] comprehensively observed HIV primary infection: 3173 individuals from four countries (Kenya, Tanzania, Thailand and Uganda) who were uninfected but at high risk for acquiring HIV were enrolled. A total of 155 acute HIV-1 infections were diagnosed during the study, of which we consider the data from 46. Antiretroviral therapy was not initiated immediately, and individuals were followed for up to 5 years after diagnosis.

Here, we take the pNLME approach and use this unique dataset to test 30 formulations of HIV primary infection models. By information criterion, we identify the most parsimonious model and use it for timing estimation, which includes a stochastic formulation of the model informed by previous stochastic viral dynamics [5,21]. An exploration of the robustness of the model shows that substantial information is gained from the whole viral load trajectory, such that the infection time from individuals with sparse data (missing upslopes) can still be estimated. Finally, the model is used to explore ideal clinical sampling schemes to balance practicality and a reasonable level of certainty in timing estimates.

## 2. Results

### 2.1. A framework for estimating infection time using viral dynamics

**Figure 1.**
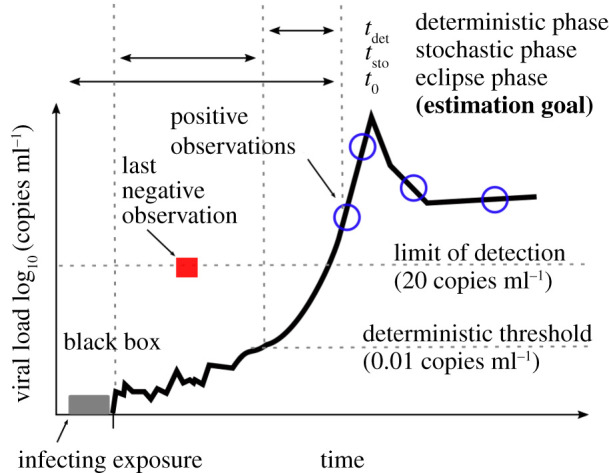
Schematic of modelling definitions. The time between infecting exposure and first positive viral load can be described in three phases. First, we recognize the possibility of an unknown but probably brief ‘black-box’ period describing localized biology that exists immediately after infecting exposure. Second, a stochastic process governs early viral expansion, starting with one or a few infected cells initiating systemic infection in the new host—and concluding when viral load reaches the deterministic threshold $\left(t_{\mathrm{sto}}\right)$. Third, a deterministic model $\left(t_{\det}\right)$ proceeds, describing the observed viral dynamics. By combining estimates for these phases, we finalize our estimate of $t_{0}$, the time between infecting exposure and first positive viral load, sometimes referred to as the eclipse period.

We assumed that HIV infection begins with an *infecting exposure*, which is the target time of estimation*.* Our estimation framework then assumed three main conceptual phases from infecting exposure through primary infection: (i) black box, (ii) stochastic, and (iii) deterministic (figure 1). We applied a separate mathematical approach to the stochastic and deterministic phases (equations and details in Methods).

The *black-box* phase immediately following the *infecting exposure* encompasses relatively unknown biology. This could include virus diffusion across mucosal barriers, creation/extinction of infection foci, target-cell trafficking to the infection site and/or viral dissemination to draining lymph nodes. Animal challenge studies and human cases where infecting exposure is well documented suggest the black-box phase is brief, from a few hours to 1 day [22–27]. Thus, we did not include a mathematical model of this period but noted this fundamental uncertainty.

Next, a few infected cells begin the process of viral replication in a *stochastic phase* with duration $t_{\mathrm{sto}}$. The stochastic phase entirely comprises viral loads below the limit of detection (20 copies ml^−1^) and permits viral extinction. Thus, a stochastic model of discrete cell/virus populations was used. Once viral loads cross the *deterministic threshold* (*t*_det_), their kinetics are approximately exponential and extinction is practically impossible. Thus, a deterministic model was used for this *deterministic phase.* We combine the results of these two models to estimate the time $t_{0}$ between infecting exposure and first positive viral load, sometimes referred to as the *eclipse phase* [5,7].

### 2.2. Experimental viral load data with statistical imputation from Aptima

We used viral load observations from the RV217 study [20], including 46 individuals out of 155 total diagnosed acute HIV-1 infections in the study. Individuals had twice-weekly HIV tests before diagnosis using the Aptima HIV-1 RNA Qualitative Assay (Hologic)—a fingerstick device testing small blood collection (0.5 ml). Once diagnosed (two Aptima-positive visits), quantitative polymerase chain reaction was used to quantify HIV RNA twice weekly in those individuals who did not initiate antiretroviral treatment (ART) and had approximately 10 study visits in the first month after diagnosis. From this cohort, we assembled viral loads from Thai and Ugandan men, women and transgender individuals. Only individuals with more than three detectable longitudinal viral load observations were included.

**Figure 2.**
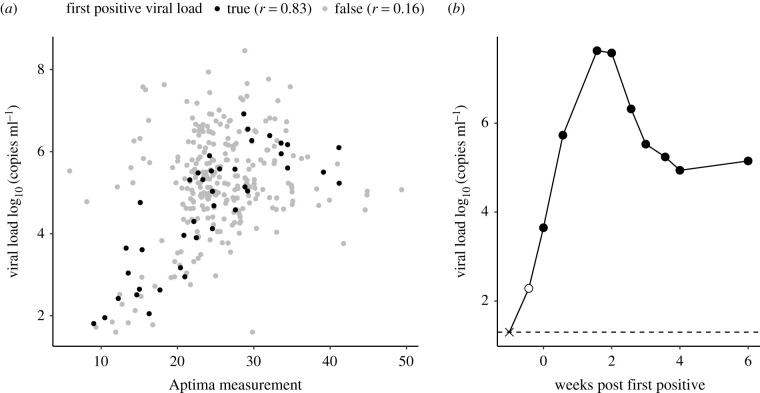
Correlation plot between Aptima measurements and viral load. (*a*) A strong linear correlation (Pearson *r* = 0.83) was found between Aptima and viral load (log_10_ HIV RNA copies per ml) at the first positive viral load (black dots). If positive samples beyond the first positive (grey dots) were included, and at higher measurements of either outcome, Aptima was less correlated to viral load. (*b*) We used diagnostic Aptima measurements prior to the first positive viral load to impute additional viral load values. Here, the filled circles indicate observed viral load measurements, the open circle indicates an Aptima imputed value and the times symbol indicates the last negative measurement.

We found that, during acute infection, Aptima and viral load were strongly correlated (figure 2*a*) and Aptima measurements could be used to impute viral load at diagnosis times for individuals without measured viral loads (figure 2*b*). Using this relationship, we imputed viral load at Aptima diagnosis for 28 participants (electronic supplementary material, figure S8), which adjusted the first positive viral load by a few days.

Several individuals were not diagnosed until later acute infection, meaning that the peak and upslope of viral load are not obviously detected. We do not exclude these individuals, instead relying on our population modelling approach and borrowing strength across the cohort to make estimates. These estimates are particularly useful because such datasets provide significant challenges to other timing estimation approaches.

### 2.3. Inference of $t_{\det}$ from a parsimonious model of the RV217 cohort data

**Figure 3.**
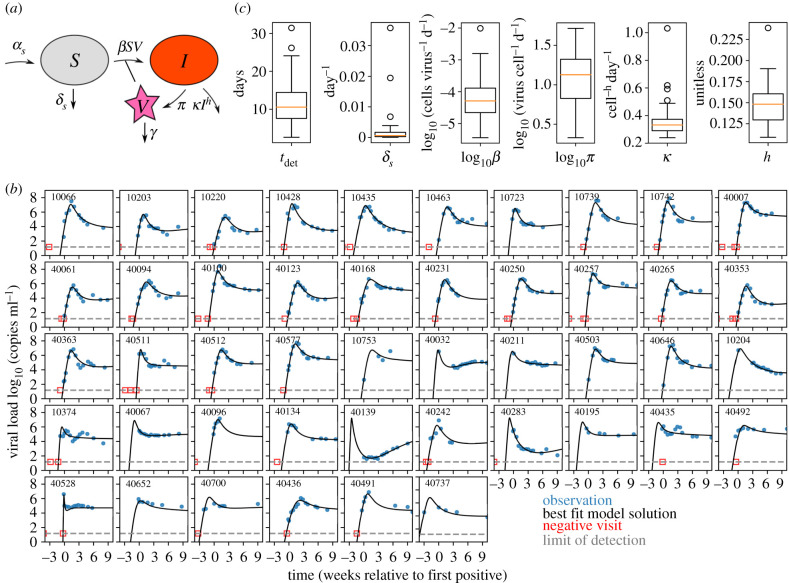
The optimal mathematical model recapitulating RV217 viral load kinetics. (*a*) We tested 30 models, which included four mechanistic models with many statistical models for each, and found that the optimal model was a variant of the canonical viral dynamics model where infected cells have a nonlinear death rate (equation (4.1)). (*b*) This model captures diverse viral load kinetics in the RV217 human study. For each individual (panel), we show viral load data (blue dots), best individual fit (black line), limit of detection (grey dashed line, 20 copies ml^−1^) and last negative visit (red square, included as censored data for fitting). By borrowing strength through the population fitting approach, the model infers peak and upslope even when those data are missing (see participant ID 40139 and 40700, for example). Imputed Aptima data are applied and one individual (last panel, 40737) had a first positive viral load shifted substantially. (*c*) Distributions of the six estimated parameters including the deterministic time.

The first step in estimating $t_{\det}$ was developing a model that best described the observed data. Thus, we selected four distinct and previously applied mechanistic models of HIV primary infection and varied their population parametrizations (the number and type of parameters estimated). This resulted in a total of 30 models (see electronic supplementary material, table S1). The four mechanistic models included the canonical viral dynamics model [28], two models recently fitted to simian–human immunodeficiency virus (SHIV)/simian immunodeficiency virus (SIV) viral dynamics [29,30] and our own simplified model based upon Holte *et al*. [31]. We found that the most parsimonious model of the RV217 cohort data (electronic supplementary material, figure S1 and table S1) includes susceptible target cells (*S*) that are born and die naturally and virus (*V*) that infects these cells and creates productively infected cells that produce viable virus (*I*). The infected cell death rate depends on their own density powered by an exponent (*h*). This term coarsely encapsulates natural cytopathic cell death during viral production, as well as innate or acquired immunity against HIV-infected cells that escalates as the number of infected cells increases (figure 3*a*; see also Methods and equation (4.1)). In this way, an explicit immune effector compartment is not needed, and the model is simplified substantially.

The model output is congruent with previous data for other model compartments. For example, it predicts a susceptible cell drop between 40% and 80% [32] (which may relate to the CD4^+^ T-cell depletion during peak viraemia [20]) and allows for the large observed inter-participant variation of viral peak (electronic supplementary material, figure S2*a*). The model also estimates that susceptible CD4^+^ T cells are long-lived and have a pool of 10^5^–10^6^ cells µl^−1^, which may include cells from the lymphoid tissue.

The best-fit model for each individual is displayed in figure 3*b*. We used pNLME modelling to estimate parameters, such that each individual has their own estimated parameters, but these estimates are constrained to be drawn from population distributions of each parameter; the population distribution is simultaneously estimated. All distributions of parameter estimates are shown in figure 3*c* and electronic supplementary material, table S2 and values are quoted for each individual in electronic supplementary material, table S3.

Across all 46 participants, the estimated deterministic time was a median of 10 days (range: 2.5–32.6). We also verified that models with comparable Akaike information criterion (AIC) (less than 10 difference from the best model AIC score) admitted similar individual values for $t_{\det}$. Summary statistics of viral load (peak and set point) were not correlated with deterministic time $t_{\det}$; rather they were strongly correlated with estimated infectivity (β), viral production rate (π) and the nonlinear death exponent (h) (electronic supplementary material, figure S2*b*). The magnitude of the first positive viral load was significantly, but not strongly, correlated with $t_{\det}\;$ (electronic supplementary material, figure S3). These results suggest that other estimated parameters are mostly independent of infection timing and that the model predictions are informative beyond upslope regression—i.e. nonlinear estimation enhances our predictive power.

### 2.4. Stochastic simulations up to the deterministic threshold

Evidence from modelling other viruses suggests that early stochastic events are linked to later deterministic kinetics [33]. For example, for cytomegalovirus (CMV) infection, extinction probabilities, duration and magnitude of transient stochastic infections are consistent with primary infection mathematical model parameters [34]. Therefore, based on the individual best-fit parameter sets, we performed stochastic simulations to determine the time window between the introduction of a single infected cell and the deterministic threshold $\left(t_{\mathrm{sto}}\right)$. Simulations were initialized with a single infected cell per microlitre I(0)=1 and at the viral-free equilibrium between susceptible cell birth and death $S(0)=\mathrm{vol}\times \alpha_{S}/\delta_{S}$. Scaling up to realistic volumes allows for a discretized stochastic simulation; vol was chosen to be 5 × 10^8^ µl, or 5 l, of blood (typical for an adult human) at approximately 100-fold concentration based on the finding that the majority of lymphocytes reside in lymphoid tissues where infection is assumed to initiate before spilling over into blood [26,35].

For each individual, the best-fit parameters of the deterministic model were used to conduct 100 stochastic simulations via the tau-leap method [36]. Because HIV transmission is a rare per coital event [37] and we are interested in infection time estimation, we conditioned upon successful infection [5] by only using simulations from stochastic runs that did not go extinct.

**Figure 4.**
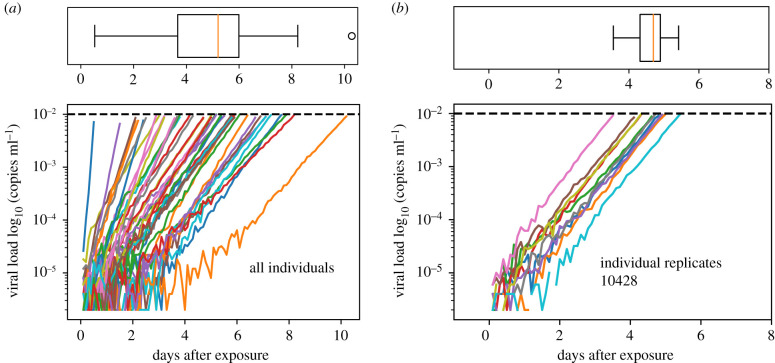
Stochastic simulations using best-fit parameter estimates from the deterministic model. Viral load kinetics until the deterministic threshold (0.01 copies ml^−1^). (*a*) A single stochastic realization for all 46 individual parameter sets with an associated boxplot of the distribution of times (over these 46 simulations) to reach the deterministic threshold. The slopes are different across individuals owing to the different parameter estimates from the deterministic models. (*b*) Ten replicate stochastic realizations for a single individual with an associated boxplot of the distribution of times (over these 10 simulations) to reach the deterministic threshold. Here, slopes are nearly identical, but, owing to the stochasticity of the simulation, the time to reach the deterministic threshold varies between 3 and 5 days. Note any discontinuities in lines are artefacts of down-sampling to reduce the image file size.

We estimated the deterministic threshold through repeated stochastic simulations, finding that a value of 0.01 copies ml^−1^ was low enough to permit rapid simulation and to sufficiently satisfy two criteria: (i) the slope of stochastic viral loads were nearly log-linear and (ii) there was effectively no chance of stochastic burn out. The simulations were halted when viral load crossed the deterministic threshold and the time to reach that viral level $\left(t_{\mathrm{sto}}\right)$ was recorded. Simulated viral loads from a single stochastic simulation of each individual are shown in figure 4*a*. The distribution of stochastic times $\left(t_{\mathrm{sto}}\right)$ is visualized above the viral load panel, indicating a slightly asymmetric time to crossing the deterministic threshold with a median of approximately 5 days in this single stochastic simulation. There is substantial variability in the slope of these viral load trajectories based on the range of parameters inferred from the deterministic model for each individual. We also performed replicate simulations for single individuals (10 replicates for participant 10428 are shown in figure 4*b*). In this case, viral load slopes are nearly identical by the time the deterministic threshold is crossed, but the early stochastic events introduce some variability in $t_{\mathrm{sto}}$. For this individual, the median time between infection and deterministic threshold was 5 days, with a total range between 3 and 5 days in these 10 simulations. In summary, viral load upslope varies highly across subjects but minimally within subjects. Variability introduced by the stochastic phase is predominantly a shift, rather than a scaling of infection time. This agrees with modelling of barcoded virus data early in infection [38].

An important parameter for these simulations is the initial number of infected cells. We show that estimates of $t_{\mathrm{sto}}$ are inversely correlated with I(0). For example, as I(0) was increased from 1, 10, 100 to 1000, the median estimate of $t_{\mathrm{sto}}$ across individuals decreased from 5 days to 1 day (electronic supplementary material, figure S4). As a result, this difficult-to-measure biological variable contributes roughly four additional days of uncertainty over three orders of magnitude. On the other hand, the variance of $t_{\mathrm{sto}}$ across individuals decreases as the initial number of infected cells increases, such that population estimates are more consistent in this regime.

### 2.5. Combining the stochastic and deterministic phases to estimate infection time

**Figure 5.**
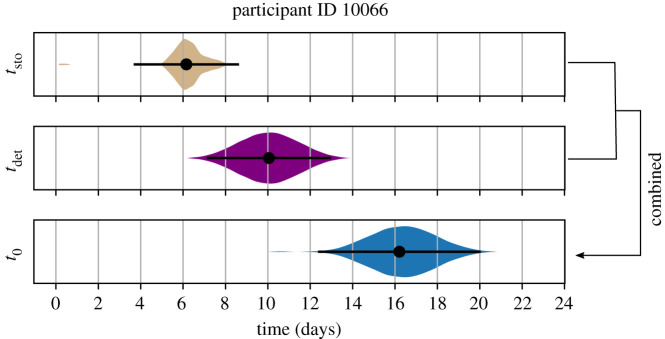
Individual estimate example. A bootstrap combination of the deterministic and stochastic estimates provides an estimate for an individual's (10066) time of infection. The stochastic time interval $\left(t_{\mathrm{sto}}\right)$ between one infected cell and the deterministic threshold (0.01 copies ml^−1^) was determined by 100 replicate stochastic simulations for that individual. Here, the mean estimate of $t_{\mathrm{sto}}$ was 6 days (dot) with a 95% uncertainty interval (lines) ranging between 4 and 9 days. The entire probability distribution is shown to illustrate skew. The time interval between the deterministic threshold and the first positive viral load was determined by the best estimate of $t_{\det}$. Here, the mean (dot, approx. 10 days) and 95% uncertainty interval (lines, ranging between 7 and 13 days) from the MCMC estimate are shown with the derived distribution. Finally, the distribution of times between infection and first positive viral load $\left(t_{0}\right)$ is calculated from 10 000 random combinations from 100 draws each of $t_{\det}$ and $t_{\mathrm{sto}}$. Our best estimate suggests this individual was infected 16 days prior to first positive viral load with the 95% uncertainty interval ranging from 12 to 20 days.

Next, we integrated the stochastic and deterministic timing estimates to complete the estimation of $t_{0}$, the time between infecting exposure and first positive viral load or the eclipse phase. As an example, we present this procedure for participant 10066 (figure 5). First, we used the best-fit parameters and performed 100 replicate stochastic simulations to estimate a distribution of $t_{\mathrm{sto}}$; the mean was approximately 6 days, and the distribution was skewed, with a 95% uncertainty interval ranging between 4 and 9 days*.* Second, we drew 100 values of $t_{\det}$ from a constructed conditional distribution using Markov chain Monte Carlo (MCMC), given the population and random effect estimates of$\;t_{\det}$ (mean 10, 95% uncertainty interval between 7 and 13 days). The infection time, $t_{0}$, and its associated 95% uncertainty interval were then calculated by creating all 100 × 100 combinations of the sum of $t_{\mathrm{sto}}$ and $t_{\det}$ from respective distributions. Estimates of $t_{\mathrm{sto}},t_{\det}$ and $t_{0}$ for this individual are presented in figure 5; we estimated that this individual's infection occurred 16 days prior to first positive viral load with the 95% uncertainty interval ranging between 12 and 20 days. This procedure was performed for all individuals.

### 2.6. Direct comparison with previously applied infection timing estimation tools

**Figure 6.**
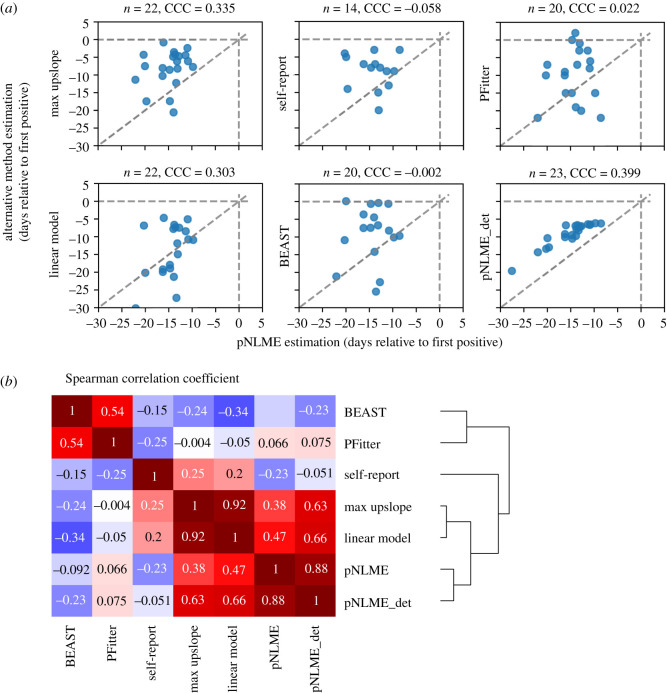
Comparisons of the pNLME approach for infection timing versus five other methods. Methods include the maximum slope of any two points on the upslope, the best log-linear regression slope (linear model), self-report from trial participants, Bayesian phylogenetic inference of the median time to most recent common ancestor (BEAST), Poisson fitter (PFitter) diversity-based estimator and our approach restricted to only the deterministic (_det) component. (*a*) Best estimate of each available individual from each method expressed as predicted infection time relative to first positive viral load (all estimates are tabulated in electronic supplementary material, table S4). Comparison sample size *n* is denoted above each panel because some approaches are constrained by features of the data (e.g. detection of upslope, sequencing characteristics). Concordance correlation coefficients (CCC) show that individual agreement is generally weak: CCC = 1 when all data lie on the line *y* = *x* (dashed diagonal line). (*b*) Hierarchical clustering by the Spearman correlation illustrates which methods are predictive of others.

Rolland *et al*. [3] used several viral load and phylogenetic inference techniques to estimate infection times using the RV217 data. These methods included the maximum slope of any two points on the upslope (max upslope), the best log-linear regression slope (linear model), self-reported entries from trial participants (self-report), Bayesian phylogenetic inference of median time to most recent common ancestor (BEAST) [39] and Poisson fitter [1] (PFitter) diversity estimator based on envelope sequences sampled at three time points in the first six months of infection. We compared all methods against one another and against our viral dynamics pNLME estimates (both full and deterministic ‘_det’; figure 6). In general, our deterministic estimates were in the same range as the other estimates. However, concordance between methods was fairly weak: concordance correlation coefficients (CCC), which score how closely data lie to the line *y* = *x*, are presented in figure 6*a*. The lack of concordance is driven by the fact that our full estimate finds infection time to be earlier than other estimates. Adjusting the initial number of infected cells from 1 to 1000 (see electronic supplementary material, figure S4), or removing the stochastic phase moves our estimates closer to previous estimates. Finally, we show correlation between full and deterministic-only pNLME (final panel in figure 6*a*) to illustrate that their estimates are not linearly related. Previous approaches were not strongly predictive of one another either (figure 6*b*). Hierarchical clustering by the Spearman correlation grouped sequence-based estimators (BEAST, PFitter) and viral dynamics estimators (max slope, linear model) with self-report diary entries falling roughly in between. pNLME was more akin to other viral dynamics approaches.

The pNLME approach had several qualitative advantages. It provided estimates for all individuals, including those with missing upslopes. It did not produce large outliers and never estimated the time of infection to be at or within a day of first positive, as max slope, BEAST and PFitter did in a few cases.

Konrad *et al*. [5] applied mathematical modelling and maximum upslope estimators on a different dataset to estimate the eclipse period between exposure and detection as 8–10 days. We did not compare directly with this approach, but their estimates agree generally with those of Rolland *et al.* [3]. For the same reasons, our results estimate a possible earlier time of infection.

### 2.7. Insensitivity of pNLME to multi-founder infections

pNLME was not strongly sensitive to multiple-founder infections (which can complicate sequence-based estimation). For example, Rolland *et al*. [3] identified some individuals in this cohort infected with multiple-founder viruses; for those infections, naive estimates of time to most recent common ancestor admitted estimates preceding the date of last negative test by many months (reflecting divergence in the transmitting partner rather than divergence after transmission).

Infection with multiple founders has been associated with higher set-point viral loads [40]. Therefore, we tested to see if our model parameter estimates were different in single versus multi-founder infections (electronic supplementary material, figure S6). We observed no obvious patterns distinguishing single and multi-founder participants and found no significant differences among our parameters (Mann–Whitney *p* > 0.1) but note the limited sample size with these data (*n* = 9 multiple founders in this set). Importantly, the deterministic time was not affected by the distinction of multiple founders.

### 2.8. Robustness to sparse data and missing viral load upslopes

An analysis of the deterministic time estimates in individuals for whom viral load upslope was not detected showed that model parameter estimates were different in these individuals (electronic supplementary material, figure S7). Since missing upslope intuitively suggests that infection was earlier before the first positive viral load, this analysis validated that pNLME does not simply predict the population average. Importantly, information was gained from post-peak kinetics that helps estimate infection time in individuals with missing upslopes.

**Figure 7.**
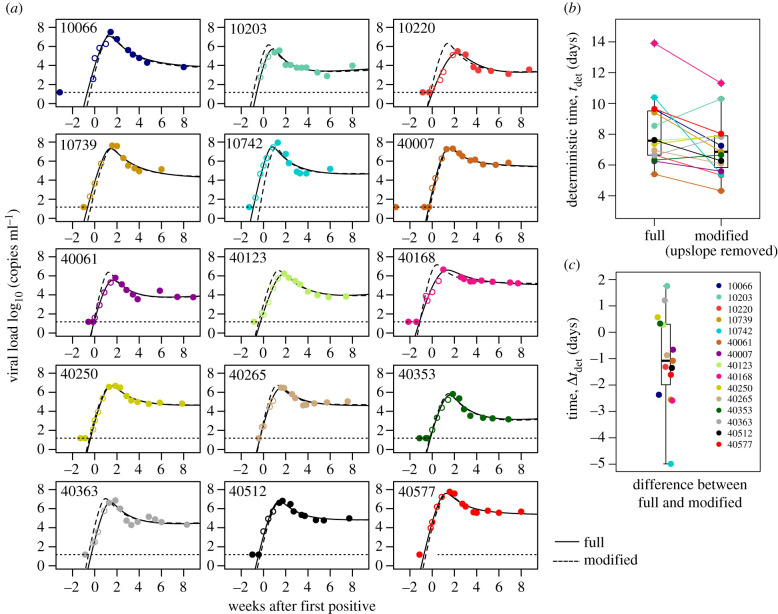
Model estimates of individuals after removing observed upslopes. (*a*) Fifteen examples of participants including their original full fit (solid line) and a modified fit after removing data from the viral load upslope (dashed line, white circles are removed data). (*b*) Estimates for deterministic times using the full and modified data, and (*c*) difference in the estimate of deterministic time between full and modified fits shows a minor bias (median less than 2 days difference).

We also validated that leaving out a participant's upslope observations did not substantially alter their timing estimate. We identified 15 participants who had at least three observations during the viral load upslope. One at a time, each of the 15 was left out (so their data did not influence the population estimate), and then the population fit from all other (*n* = 45) individuals was used to fit a modified dataset for the left-out participant with removed upslope observations (figure 7). The modified fits were reasonably similar by visual assessment (figure 7*a*). The median deterministic time for these 15 participants decreased from 7.5 to 6.5 days, meaning that estimated $t_{\det}$ in individuals lacking upslope data tends to be a slight underestimation (figure 7*b*). The individual errors (differences in pairs from figure 7*b*) indicated a loss of precision (range: −5 to 2 days; interquartile range: −2 to 0 days) and a median bias of −1 day (figure 7*c*).

### 2.9. Additional sources of uncertainty

In our model timing schematic (figure 1), we consider the possibility of non-mechanistic delays in viral load captured by the black-box period. This period between exposure and viral dynamics has not been explored in detail in the human model. Macaque models of SIV and SHIV suggest less than 1 day between exposure and viral replication kinetics [22,24,25,41]; however, in addition to possible differences between HIV and SIV/SHIV biology, these experiments often have large inoculum sizes that could make comparison difficult [42,43]. With that in mind, we assume that time increases from non-mechanistic processes like anatomic distribution are minor relative to the approximately 6 day $t_{\mathrm{sto}}$ period predicted through our approach.

Another consideration is that infected cells do not immediately produce virus. An additional 1 day ‘cellular eclipse phase’ is included in some HIV viral dynamics models [44] (including that of Konrad *et al*. [5]). Therefore, we performed an analysis estimating $t_{\mathrm{sto}}$ using a model with cellular eclipse, a 1 day waiting period before infected cells become productively infectious. Intuitively, this process roughly shifted the distribution of $t_{\mathrm{sto}}$ by 1 day (see electronic supplementary material, figure S5).

### 2.10. Proof-of-concept study on synthetic data with realistic study protocols

**Figure 8.**
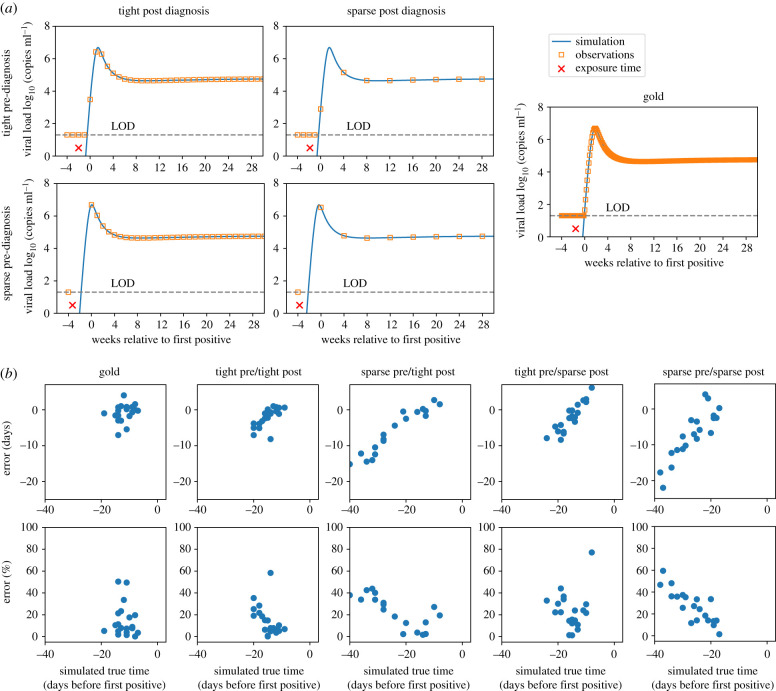
Accuracy of timing tested on simulated viral load data with sparse and tight study sampling. We simulated randomly occurring infections using individual parameter sets and sampled according to four different theoretical study protocols, tight (weekly) and sparse (monthly) before and after diagnosis. If viral load was above the limit of detection (LOD, 20 copies ml^−1^) of an observation, this was called the first positive (or diagnosis) and post-diagnosis sampling began. (*a*) Four examples of each study protocol: combinations of tight and sparse, pre- and post diagnosis. True infection is denoted with the red times symbol, simulated viral load with the blue line and observations given the protocol with the orange squares. (*b*) Twenty individuals were simulated with each protocol, and pNLME inference was performed on those data. The accuracy of the estimated $t_{0}$ compared with the true infection time is shown as the error in days (difference between pNLME estimated and true time) and per cent error (error relative to true time × 100%) for each sampling protocol. Low error, therefore, indicates estimates that agree, and per cent error illustrates how estimates become more biased (relative to other estimates) as the time between detection and true infection time increases.

To assess how sampling intervals affect the accuracy of pNLME timing estimates, we performed a simulation study. We simulated viral loads from 20 randomly chosen RV217 participants and sampled these viral loads with five different theoretical protocols. We refer to ‘gold’ as daily, ‘tight’ as weekly and ‘sparse’ as monthly sampling visits (every four weeks). Infection was assumed to occur uniformly (no relation to study visits). If viral load was above 20 copies ml^−1^ at a visit the first positive (or diagnosis) was recorded and measurements occurred subsequently. In figure 8*a*, we illustrate an example of each protocol with tight and sparse samplings before and after diagnosis. We estimated infection timing on these synthetic data by assuming that each synthetic dataset was a new individual whose parameters could be drawn from the population distributions derived from the RV217-trained model. Their newly specified parameters were applied to the stochastic modelling step to complete the timing estimate.

Figure 8*b* shows the absolute error (days difference between truth from the synthetic data and inferred $t_{0}$ from pNLME applied to those data) and the per cent error: (true − inferred)/true × 100%. Gold and tight/tight sampling predictably admitted the lowest errors. Error was generally negative (with some exceptions for sparse post), meaning that true simulated infection time was closer to first positive than estimated.

All schemes other than gold had an obvious bias. Absolute and per cent error were higher in individuals for whom true infection time was earlier. This means that uncertainty rises with estimation time further from first positive. Put another way, our confidence decreases as the estimator projects further into the past—an intuitively satisfying, albeit challenging, finding. The linearity of the bias hints that it might be corrected. However, it may be an artefact of our synthetic data exercise, so we opted not to follow through with any correction. Rather, we focus on individuals who appear to have been infected within 20 days since first positive. For all sampling schemes, error on these estimates has a median of ±10 days. An important result is that sparse sampling after diagnosis was less detrimental than sparse sampling before diagnosis, because of the growing uncertainty with time and the likelihood of missing upslope, peak and downslope.

## 3. Discussion

Estimating infection time is critical for infection-prevention trials. Determination of drug concentration at the precise time of breakthrough infections is a key step to defining protective levels. Here, we have developed an approach to estimate HIV infection time using a viral dynamics modelling framework applied to data from the RV217 cohort, an acute HIV infection study with exquisitely fine viral load sampling.

Through extensive information-theoretic model selection using pNLME models, we discovered a relatively simple mechanistic model that accurately fits to all viral loads. This model was used in a deterministic and stochastic framework to estimate the infection time of all study participants. This model could additionally be used in a more general setting to simulate future trials.

In relation to other previously used timing methods, our approach has several advantages. It allows estimation in individuals without observed viral upslope or even viral peaks. It is relatively insensitive to multiple-founder infections, a possible challenge for sequence-based methods.

Although individual estimates were not highly concordant with previous estimates for the same dataset, averages agreed between other methods and our deterministic estimate. However, our additional stochastic component drives our estimates to be earlier, extending the eclipse time. Concordance of our model was strongest against estimates using other viral load approaches (at best CCC were approx. 0.4), intermediate against self-report diaries and weakest against estimates using sequence-based approaches. Therefore, a mosaic approach may be optimal, developing uncertainty intervals across all methods. A future solution would be to model sequences and sequence evolution explicitly and apply both types of data [45].

While the true time of infection cannot be known other than in challenge experiments, we verified that our framework is self-consistent. That is, it accurately estimates infection time on simulated data from the same mechanistic model. The ‘sparse/sparse’ case was chosen because it represents a comparable (though slightly sparser) sampling scheme to that of the broadly neutralizing antibody-mediated prevention (AMP) studies [46,47]. In that study, drug infusions occur every eight weeks and diagnostic visits occur every four weeks; after first positive viral load (week 0) visits occur at weeks 2, 4, 8, 12 and 24 [46,48,49]. Thus, given the additional assumption that HIV dynamics in participants in the AMP study are comparable to participants in RV217, we expect that our approach would provide reasonably accurate estimates for individuals who appear to have been infected within three weeks of the first positive visit (95% uncertainty interval approx. 5 days). Estimates become worse for earlier infections, but fortunately earlier infections should be less likely as drug concentrations are higher. In any case, this challenge is not unique to our approach. We do show that tighter sampling after diagnosis does not drastically improve accuracy. Indeed, two important implications for trial design are that (i) timing accuracy is more improved by tighter sampling prior to rather than post diagnosis and (ii) high-sensitivity (e.g. Aptima) testing is crucial to avoid the challenging situation when a recently infected individual is not diagnosed at a study visit. We found the Aptima assay measurements advertised as qualitative were highly correlated with viral loads such that missing early viral loads may be imputed from Aptima with relatively high confidence.

There are several open questions for the framework's validity. First, it remains unknown, and will be extremely hard to test, whether early HIV dynamics can be described by the same mechanistic model as deterministic viral dynamics. However, in CMV transmission, the probability of infection has been related to post-infection viral kinetics, suggesting that stochastic behaviours may be linked to subsequent deterministic kinetics [34]. Second, acute HIV infection probably encompasses localized exposure and viral transport/diffusion through anatomical barriers before initiating systemic infection [50]. The duration of this period is unknown but is likely to be short [26] and it could be modelled as an additional source of uncertainty. It is not clear if any timing method can directly account for this period; for example, what is ultimately determined as a founder sequence may be a virion that has already passed through this step. Third, our choice of the initial simulation conditions I(0) does inversely correlate with the time of infection. That is, if we assume viral infection begins at a lower level, our estimates of infection time are pushed earlier. However, across what we consider to be a plausible range of 1–1000 infected cells initiating infection, the median estimation varies by 4 days (electronic supplementary material, figure S4). Interestingly, Rolland *et al*. [3] found that an initial viral load of 1 copy ml^−1^ gave the best estimates for a log-linear regression model of non-human primate infection where the date of infection was known perfectly. One might, therefore, choose this value for the deterministic threshold, but the translation of this estimate is complicated by the challenge virus and the animal inoculation process—typically a high viral load inoculation is used to ensure ultimate infection. Therefore, this experimental set-up may require a higher initial condition for a mathematical model than a typical human exposure, which is known to not always cause infection.

Viral dynamics models exist for many viruses [51,52] and, thus, our approach is immediately applicable. Recently, a similar method was applied to estimate the time of SARS-COV-2 infection [53]. In future work, we plan to explore modifications due to preventive interventions, such that infection timing, and, therefore, protective levels, can be better estimated for AMP and other coming HIV prevention trials.

## 4. Methods

### 4.1. Model selection

We explored four main different mechanistic models. Three have previously been used to model primary HIV infection viral loads and the other was inspired by a previously used model for HIV decay after ART initiation. For each mechanistic model, we varied assumptions about the fixed and estimated parameters, leading to a total of 30 models tested against the RV217 viral load data. To determine the most parsimonious model, we fitted each model to the data and used an information criterion to rank their support from the data [54]. For each of the 30 models, we performed 15 repeated optimizations of model fit using the stochastic approximation expectation-maximization (SAEM) algorithm. From each optimization, we calculated the log-likelihood (logL) to quantify model fit. Then, we computed the AIC and Bayesian information criteria (BIC) for the model with highest likelihood $\left(L_{\max}\right)$ among the 15 assessments: $\mathrm{AIC}=-2\log L_{\max}+2m$, $\mathrm{BIC}=-2\log L_{\max}+\log(n)m$, where *m* is the number of parameters estimated and *n* is the total number of data points. Two models were considered similarly supported by the data if the difference between their AIC and/or BIC was less than 2 [54]. Electronic supplementary material, table S1 contains the equations for each mechanistic model, the statistical assumptions and the AIC and BIC values. Electronic supplementary material, figure S1 shows the distribution of likelihoods from the repeated stochastic optimizations, as well as the median and best AIC value.

### 4.2. Population nonlinear mixed-effects model fitting

We modelled the plasma viral load using a nonlinear mixed-effects approach (pNLME). In this approach, an observed plasma viral load for individual *i* at time j is modelled as $\log_{10}V_{ij}=f_{V}(t_{ij},\theta_{i})+\epsilon_{V}$. Here, $f_{V}$ is the log_10_ viral load from the solution of the selected mechanistic model (see equation (4.1)). $\theta_{i}$ is the m-length vector of parameters for each study participant and $\epsilon_{V}∼N(0,\sigma_{V}^{2})$ is the estimated measurement error for the viral load with standard deviation $\sigma_{V}$. Note this implies that viral load noise is lognormally distributed. The pNLME approach assumes that each individual-specific parameter $\theta_{ik}$ is drawn from a probability distribution with the median or fixed effects $\theta_{k}^{\mathrm{pop}}$ and random effects $\eta_{ik}∼N(0,\Omega )$, where $\Omega =\Omega_{kl}$ is the covariance matrix (admitting the parameter variance when k=l and the covariance among parameters when k≠l). Because they are extremely sensitive parameters and potentially logarithmically different among individuals, we modelled parameters β and π as $\log_{10}\theta_{ik}=\theta_{k}^{\mathrm{pop}}+\eta_{ik}$. The remaining parameters were modelled as $\theta_{ik}=\theta_{k}^{\mathrm{pop}}\exp(\eta_{ik})$.

For each model, we obtained the maximum-likelihood estimation (MLE) of the measurement error standard deviation $\sigma_{V}$, the fixed effects vector $\theta^{\mathrm{pop}}$ and the elements of matrix Ω using the SAEM algorithm embedded in the Monolix software (www.lixoft.eu). For all model fits, we assumed $t_{ij}=0$ as the time of the first positive viral load. However, we defined the initial value as the time $-t_{\det}$ when $V(-t_{\det})=0.01$ copies ml^−1^. We fixed other initial values as $S(t_{\det})=(\alpha_{s}/\delta_{S})$ cells µl^−1^ and $I(t_{\det})=(\gamma V(-t_{\det}))/\pi$ cells µl^−1^. As per Ramratnam *et al*. [55], we fixed parameter $\gamma =23\;{\mathrm{day}}^{-1}$. We estimated the remaining parameters of the mechanistic model including $t_{\det}$. Individual parameters were selected using the mode of the conditional distribution $p(\theta_{i}|V_{ij};\theta_{\mathrm{MLE}}^{\mathrm{pop}},\Omega_{\mathrm{MLE}})$ constructed by the MCMC algorithm in the Monolix software. The conditional distribution of $t_{\det}$ for each study participant was used to compute the time of infection $t_{0}$ (see figure 5).

### 4.3. Most parsimonious mathematical model

The set of ordinary differential equations for the model that was selected by this approach was the Holte/Cardozo model (model 2 h in electronic supplementary material, table S1). This is a slightly modified basic viral dynamics model that uses a nonlinear death term and tracks the concentration (cells ml^−1^) of HIV-susceptible cells *S*, infected cells *I* and plasma viral load *V* (viral RNA copies ml^−1^). The equations are written

4.1 $$\begin{array}{rl} \partial_{t}S & =\alpha_{S}-\delta_{S}S-\beta SV,\\ \partial_{t}I & =\beta SV-\kappa I^{h+1}\;\\ \mathrm{and}\;\;\;\partial_{t}V & =\pi I-\gamma V-\beta SV, \end{array}$$

where $\partial_{t}$ denotes the time derivative. This model requires eight free parameters $\theta_{i}=(\alpha_{S},\delta_{S},\beta ,k,h,\pi ,\gamma ,t_{\det})$. The parameters are $\alpha_{S}$ (cells μl^−1^ d^−1^) the constant growth rate of susceptible cells, $\delta_{S}$ (day^−1^) the death rate of susceptible cells and β (μl virus^−1^ d^−1^) a mass-action viral infectivity. The viral production rate is π (virions cell^−1^ d^−1^), and γ (day^−1^) is the clearance rate of virus. The death and killing of infected cells is governed by the rate of κ (cells^−h^ d^−1^), with the exponential factor *h* adjusting the density-dependent death rate. This approach coarsely approximates adaptive immunity such that higher numbers of infected cells engender faster killing.

### 4.4. Stochastic simulation scheme

We adapted the ordinary differential equation system equation (4.1) to a stochastic simulation [49]. Our implementation in Python, which employs the τ-leap approach [36], is publicly available. A time interval Δt = 0.0001 days is chosen for step size, in which a Poisson number of each transition type occurs. Initial conditions are changed to discrete values by multiplying by a volume. We choose volume v= 10^8^ µl based on the observation that there is approximately 1–10 l of blood in an adult human and that there are approximately 10–100 times more T cells in lymph tissue than in blood. A single infected cell I(0)=1 is assumed to initiate infection (see sensitivity analyses in electronic supplementary material, figure S4 for varying this assumption). No viral particles are present V(0)=0 but are produced rapidly after starting the simulation. The initial number of susceptible cells is calculated from the viral-free equilibrium $S(0)=\alpha_{S}v/\delta_{S}$.

We write the rules of the stochastic system to illustrate the transitions in each state variable (arrows), given the rate of that transition (denoted above the arrow):

4.2 $$\begin{array}{rl} & S\overset{\alpha_{S}v}{\to }S+1,\\ & S\overset{\delta_{S}S}{\to }S-1,\\ & S,\;V\overset{\beta SV/v}{---\to }S-1,\;I+1,\;V-1,\\ & I\overset{\kappa I{\left(I/v\right)}^{h}\;}{---\to }I-1,\\ & I\overset{\pi I}{\to }I-1,\;V+1\\ \;\mathrm{and}\; & V\overset{\gamma V\;}{\to }V-1. \end{array}$$

### 4.5. Leave-one-out analysis

To assess the robustness of pNLME estimation to missing viral upslope data, we performed a leave-one-out analysis predicting infection time on participants with and without inclusion of viral load data prior to peak (upslope). We identified 15 participants with at least three measurements in the upslope. For each, we created a modified dataset by removing all pre-peak viral load measurements—leaving last negative and measurement starting at peak viral load. For each modified participant's viral load, the model was refitted (leading to 15 refits of the model to data without a viral upslope). Then, each refitted model was used to predict each participant's infection time. Population-level fixed effects were compared between the full data model and the leave-one-out models to show model robustness to individuals with upslope data. Individual-level estimates of $t_{\det}$ were then compared to identify bias that may be introduced by missing upslope data.

### 4.6. Aptima and viral load correlation analysis

The Aptima HIV-1 RNA Qualitative Assay is an *in vitro* nucleic acid assay for detecting HIV in human plasma and serum. It was used as the primary diagnostic assay in the RV217 study [20]. Although the Aptima assay is advertised as ‘qualitative’, we found a strong relationship between Aptima value and viral load (figure 2). Linear regression was used to predict first positive viral load (log_10_ copies ml^−1^) with concurrent Aptima. Both untransformed and log-transformed Aptima values were tested (electronic supplementary material, figure S8*a*). One participant was removed from the model because of an Aptima measurement of 3, an outlier more than twofold lower than the next lowest value. The relationship was less clear at high values of Aptima, suggesting a saturation effect where quantitative agreement with viral load disappears. Thus, we repeated linear regression with different upper bounds of Aptima and determined the optimum value by minimizing residual mean square error of viral load prediction (electronic supplementary material, figure S8*b*). We found the best model used raw Aptima measurements as the input with an upper bound of 34 (electronic supplementary material, figure S8*b,c*). This model was applied to the data to impute first positives for participants where Aptima was measured for diagnosis without viral load (electronic supplementary material, figure S8*d*).

## Data accessibility

All code and data in the paper are available from the authors, and custom Python scripts for simulation and plotting are available at https://github.com/dbrvs/AMP-timing.

The data are provided in the electronic supplementary material [56].
